# Eunicellin-Based Diterpenoids, Hirsutalins N–R, from the Formosan Soft Coral *Cladiella* *hirsuta*

**DOI:** 10.3390/md12052446

**Published:** 2014-04-30

**Authors:** Tzu-Zin Huang, Bo-Wei Chen, Chiung-Yao Huang, Tsong-Long Hwang, Chang-Feng Dai, Jyh-Horng Sheu

**Affiliations:** 1 Department of Marine Biotechnology and Resources, National Sun Yat-sen University, Kaohsiung 804, Taiwan; E-Mails: slime112229@gmail.com (T.-Z.H.); a6152761@yahoo.com.tw (B.-W.C.); betty8575@yahoo.com.tw (C.-Y.H.); 2 Graduate Institute of Natural Products, Chang Gung University, Taoyuan 333, Taiwan; E-Mail: htl@mail.cgu.edu.tw; 3 Institute of Oceanography, National Taiwan University, Taipei 112, Taiwan; E-Mail: corallab@ntu.edu.tw; 4 Frontier Center for Ocean Science and Technology, National Sun Yat-sen University, Kaohsiung 804, Taiwan; 5 Graduate Institute of Natural Products, Kaohsiung Medical University, Kaohsiung 807, Taiwan; 6 Department of Medical Research, China Medical University Hospital, China Medical University, Taichung 404, Taiwan; 7 Asia Pacific Ocean Research Center, National Sun Yat-sen University, Kaohsiung 804, Taiwan

**Keywords:** soft coral, *Cladiellahirsuta*, eunicellins: cytotoxic activity, anti-inflammatory activity

## Abstract

New eunicellin-type hirsutalins N–R (**1**–**5**), along with two known eunicellins, (**6** and **7**) were isolated from the soft coral *Cladiella* *hirsuta*. The structures of the metabolites were determined by extensive spectroscopic analysis. Cytotoxic activity of compounds **1**–**7** against the proliferation of a limited panel of cancer cell lines was measured. The *in* *vitro* anti-inflammatory activity of compounds **1**–**7** was evaluated by measuring their ability in suppressing superoxide anion generation and elastase release in fMLP/CB-induced human neutrophils.

## 1. Introduction

**Chart 1:**
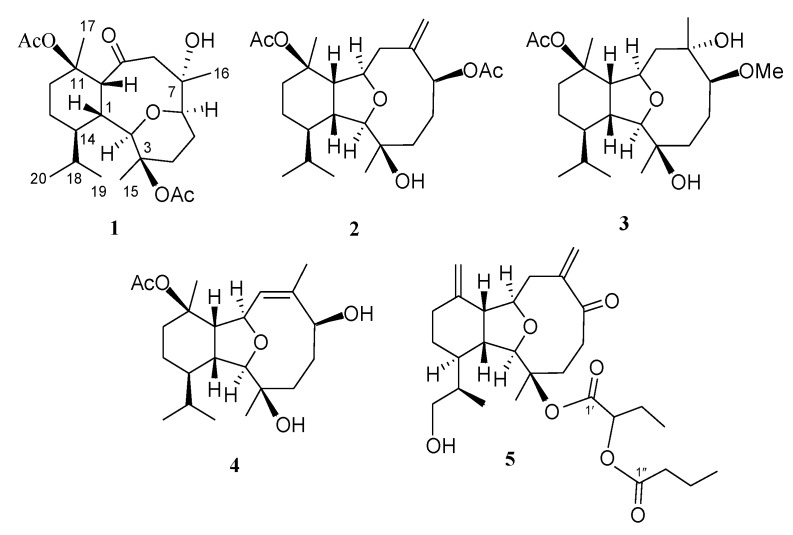
Structures of metabolites **1**–**5**.

**Chart 2:**
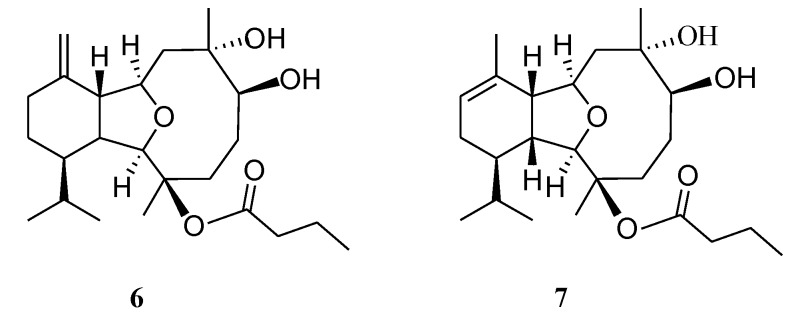
Structures of metabolites **6** and **7**.

The chemical investigations on soft corals of the genus *Cladiella* and *Klyxum* [1,2,3,4,5,6,7,8,9,10,11,12,13,14,15,16,17,18,19,20,21,22,23,24,25,26,27,28,29,30] have afforded several eunicellin-based diterpenoids, of which many have been shown to exhibit interesting bioactivities [8,10,11,12,13,14,15,16,17,18,19,20,21,22,23,24,25,26,27,28,29,30]. Our recent chemical study of a Taiwanese soft coral *Cladiella* *hirsuta* has led to the discovery of 13 eunicellin-based diterpenoids hirsutalins A–M [29,30] and seven steroids hirsutosterols A–G [31] some of which have been found to possess cytotoxic [29] and anti-inflammatory activities [29,30]. In this paper we further report the isolation of five new eunicellin-based compounds, hirsutalins N–R (Chart 1), along with two known compounds, (1R*,2R*,3R*,6S*,7S*,9R*,10R*,14R*)-3-butanoyloxycladiell-11(17)-en-6,7-diol (**6**) [6], and hirsutalin E (**7**) [29] from *C*. *hirsuta* (Chart 2). The structures of new compounds were determined by extensive spectroscopic analysis. Cytotoxicity of **1**–**7** against a limited panel of cancer cell lines and their anti-inflammatory activity, determined by their ability to inhibit the generation of super oxide anion and elastase release in *N*-formyl-methionyl-leucylphenylalanine/cytochalasin B(fMLP/CB)-induced human neutrophiles, were studied in order to discover bioactive compounds for future new drug development.

## 2. Results and Discussion

**Figure 1:**
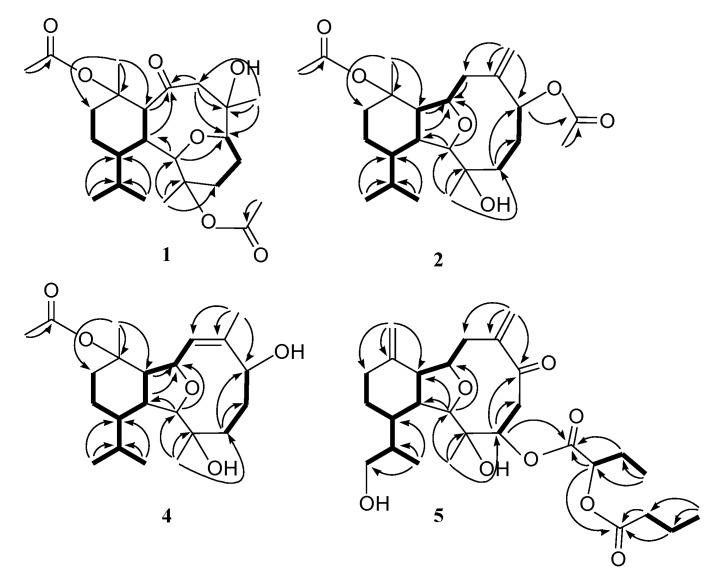
COSY and HMBC correlations for **1**, **2**, **4** and **5**.

**Figure 2:**
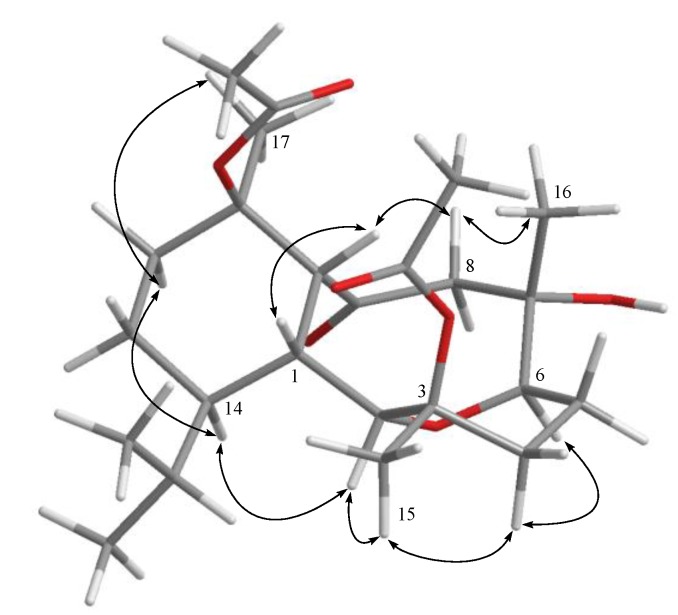
Key NOESY correlations for 1.

Hirsutalin N (**1**) was isolated as a colorless oil. The HRESIMS (*m/z* 461.2518) of **1** established a molecular formula of C_24_H_38_O_7_. The IR spectrum of **1** showed the presence of hydroxy and carbonyl groups from absorptions at 3451 and 1733 cm^−1^, respectively. The ^13^C NMR of **1** exhibited 24 carbon signals as expected which were found to be similar to these of a known metabolite hirsutalin I (**8**, Chart 3) [30], the difference being that the hydroxymethyl group attached at C-18 in hirsutalin I was replaced by a methyl group in **1**. This was confirmed by ^1^H NMR spectrum of **1** which shows the presence of two isopropyl methyls at δ 0.73 (d, *J* = 7.2 Hz) and 0.97 (d, *J* = 7.2 Hz) (Table 1). Also, NMR data revealed that the *n*-butanoyloxy group at C-3 in **8** was replaced by an acetoxy group in **1**. Key HMBC correlations from H-2 to C-6; H-1, H_2_-8, and H-10 to C-9; H_3_-15 to C-2, C-3 and C-4; H_3_-16 to C-6, C-7 and C-8; H_3_-17 to C-10, C-11 and C-12; and both H_3_-19 and H_3_-20 to C-14 and C-18, permitted the assembly of the carbon skeleton of **1**. Based on above results and HMBC correlations (Figure 1), the planar structure of **1** was established. Further, comparison of the NOE correlations of **1** (Figure 2) with those of hirsutalin I, the relative configuration of **1** was thus determined to be the same.

**Table 1:** NMR spectroscopic data for hirsutalins N–P (**1**–**3**).

1			2		3	
Position	δ_C_, mult*.* ^a,b^	δ_H_ ( *J* in Hz) ^c^	δ_C_, mult. ^a,b^	δ_H_ ( *J* in Hz) ^c^	δ_C_, mult. ^a,b^	δ_H_ ( *J* in Hz) ^c^
1	49.6, CH	2.55, dd (12.0, 4.4)	41.4, CH	2.25, m	41.9, CH	2.18, m
2	78.0, CH	3.80, s	91.3, CH	3.56, s	90.8, CH	3.56, s
3	81.3, C	-	74.0, C	-	74.7, C	-
4	27.7, CH_2_	1.36, m	34.9, CH_2_	1.75, m	41.0, CH_2_	1.83, m
	-	2.92, dd (11.8, 4.4)	-	-	-	-
5	20.6, CH_2_	1.34, m	32.0, CH_2_	1.99, m	25.7, CH_2_	1.98, m
	-	1.66, m	-	-	-	-
6	80.4, CH	3.82, dd (11.4, 6.0)	76.4, CH	5.19, dd (12.0, 6.0)	90.8, CH	4.07, m
7	85.4, C	-	149.0, C	-	76.6, C	-
8	49.5, CH_2_	2.00, d (12.0)	41.4, CH_2_	3.12, dd (13.6, 6.0)	47.0, CH_2_	1.73, m
	-	2.78, d (12.0)	-	2.47, d (13.6)	-	2.30, dd (12.8, 11.6)
9	211.4, C	-	78.3, CH	4.09, dd (11.2, 6.0)	75.6, CH	4.07, m
10	55.2, CH	4.14, dd (4.4, 2.0)	46.4, CH	2.95, dd (11.2, 7.2)	54.4, CH	2.82, t (7.6)
11	83.3, C	-	82.3, C	-	82.9, C	-
12	31.4, CH_2_	2.10, m	32.5, CH_2_	1.43, m	30.5, CH_2_	1.38, m
	-	2.24, m	-	2.24, m	-	2.40, m
13	19.3, CH_2_	1.61, m	18.2, CH_2_	1.34, m	17.7, CH_2_	1.20, m
	-	1.25, m	-	1.45, m	-	1.40, m
14	36.5, CH	1.98, m	42.8, CH	1.20, m	42.6, CH	1.22, m
15	23.6, CH_3_	1.53, s	27.4, CH_3_	1.19, s	30.3, CH_3_	1.16, s
16	22.9, CH_3_	1.13, s	118.3, CH_2_	5.29, s	23.8, CH_3_	1.16, s
	-	-	-	5.53, s	-	-
17	24.3, CH_3_	1.45, s	25.5, CH_3_	1.52, s	24.5, CH_3_	1.46, s
18	27.2, CH	1.87, m	27.9, CH	1.80, m	29.1, CH	1.71, m
19	14.5, CH_3_	0.73, d (7.2)	15.0, CH_3_	0.78, d (6.8)	15.0, CH_3_	0.78, d (6.8)
20	21.7, CH_3_	0.97, d (7.2)	21.8, CH_3_	0.94, d (6.8)	21.8, CH_3_	0.94, d (6.8)
3-OAc	22.4, CH_3_	2.00, s	-	-	-	-
	169.7, C	-	-	-	-	-
11-OAc	22.3, CH_3_	2.19, s	22.6, CH_3_	2.00, s	22.6, CH_3_	2.00, s
	170.1, C	-	170.3, C	-	170.2, C	-
6-OAc	-	-	21.4, CH_3_	1.99, s	-	-
	-	-	170.5, C	-	-	-
6-OMe	-	-	-	-	57.1, CH_3_	3.37, s

^a^ Spectra recorded at 100 MHz in CDCl_3_; ^b^ multiplicity deduced from DEPT; ^c^ spectra recorded at 400 MHz in CDCl_3_.

**Figure 3:**
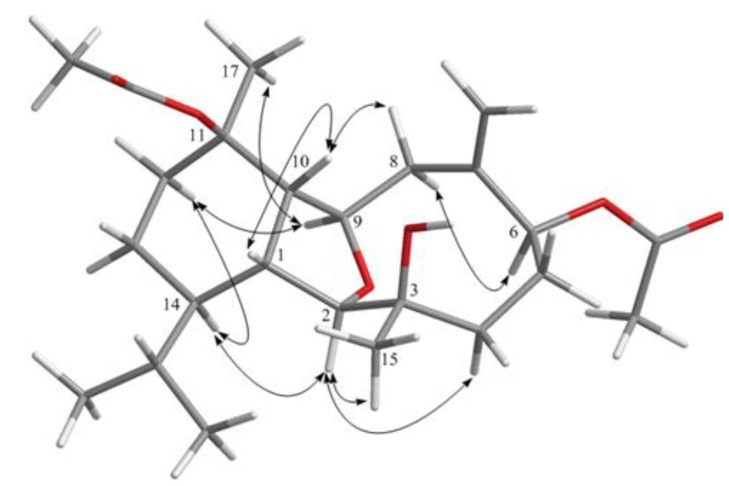
Key NOESY correlations for 2.

Hirsutalin O (**2**) was also afforded as a colorless oil. Compound **2** has a molecular formula C_24_H_38_O_6_, as determined by HRESIMS. In comparing NMR data of **2** with those of the known compound simplexin A (**9**, Chart 3) [11], it was found that the *n*-butanoyloxy group at C-3 and the hydroxy group at C-6 in simplexin A (**9**) were replaced by a hydroxy group and acetoxy group in **2**, respectively, as confirmed by the downfield shift of C-3 (δ_C_ 81.3) of **1**, relative to that of **2** (δ_C_ 74.0), and the HMBC connectivity from H-6 (δ 5.19) to the carbonyl carbon resonating at δ 170.5 (C) (Table 1). The relative configuration of **2** was confirmed to be the same as that of **9** by analysis of NOE correlations (Figure 3).

The new eunicellin, hirsutalin P (**3**), has a molecular formula C_23_H_40_O_6_ as determined by HRESIMS. The spectroscopic data (IR, ^1^H NMR, and ^13^C NMR) of **3** were similar to those of a known one, klysimplex G (**10**, Chart 3) [12], except that the acetoxy group at C-3 and the hydroxy group at C-6 in **10** were replaced by a hydroxy group and methoxy group, respectively, in **3**. The similar ^1^H NMR data and the analysis of NOE correlations of **3** further revealed the same relative configuration of both compounds. Thus, the structure of **3** was established.

**Figure 6:**
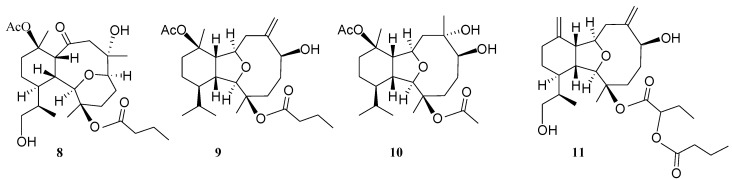
Structures of known compounds **8**–**11**.

Hirsutalin Q (**4**) was obtained as a colorless oil and exhibited a molecular formula C_22_H_36_O_5_. IR absorptions of **4** showed the presence of hydroxy and carbonyl groups at 3421 and 1724 cm^−1^, respectively. The NMR spectroscopic data revealed the presence of a trisubstituted double bond (δ_H_ 5.28, s, 1H; δ_C_ 128.4, CH and 139.4, C) (Table 2). One ester carbonyl (δ_C_ 170.2) was assigned from the ^13^C NMR spectrum and was HMBC correlated with an acetate methyl (δ_H_ 1.99 s). The chemical shift of H_3_-15 at *δ* 1.18 indicated the presence of a hydroxy group substitution at C-3, the same as that in compounds **2** and **3**. The presence of an acetoxy group at C-11 could be seen from the more downfield shift of H_3_-17 (δ 1.53), in comparison with that of H_3_-15 (δ 1.18). The planar structure of metabolite **1** was elucidated by analysis of COSY and HMBC correlations (Figure 1). The *Z* geometry of the double bond at C-7 and C-8 was evidenced by the presence of NOE correlation between H-8 and H_3_-16. In the NOESY spectrum of **4**, observation of the NOE correlation between H-1 with H-10 suggested that H-1 and H-10 are β-oriented. Also, correlations between H-2 with both H-14 and H_3_-15; H-9 with both H-14 and H_3_-17; and H-6 with H_3_-15 suggested that all of H-2, H-6, H-9, H-14, H_3_-15 and H_3_-17 are α-oriented. Thus, the structure of diterpenoid **4** was established.

A structurally-related metabolite, hirsutalin R (**5**), was also isolated as a colorless oil with a molecular formula of C_28_H_42_O_7_. Two ester carbonyl carbons (δ_C_ 169.0 and 173.5) were correlated in the HMBC spectrum with the methine proton (H-2′, δ_H_ 4.76 t, *J* = 6.8 Hz) of a 2-butyryloxybutanoate unit. Moreover, the ^13^C NMR spectroscopic data (Table 2) of **5** showed the presence of two 1, 1-disubstituted carbon–carbon double bonds (δ_C_ 147.7 (C) and 118.4 (CH_2_); 145.2 (C) and 111.6 (CH_2_)). Comparison of the NMR data of **5** with those of hirsutalin C (**11**, Chart 3) [29] revealed that the only difference between both compounds is the replacement of the hydroxy group in hirsutalin C by a ketone (δ_C_ 206.5) at C-6 in **5**. The absolute configuration of hirsutalin A [29] and hirsutalin J [30] have been completely assigned based on NOE correlations and Mosher’s method. Compounds **1**–**5** are likely in the same enantiomeric series as hirsutalin A and hirsutalin J, based on a shared biosynthetic pathway. Thus, these compounds are suggested to possess the absolute configurations as shown in formula **1**–**5**.

**Table 2:** NMR spectroscopic data for hirsutalins Q and R (**4** and **5**).

4			5	
Position	δ_C_, mult. ^a,b^	δ_H_ ( *J* in Hz) ^c^	δ_C_, mult. ^a,b^	δ_H_ ( *J* in Hz) *^c^*
1	40.9, CH	2.35, m	45.0, CH	2.25, m
2	90.8, CH	3.57, s	90.8, CH	3.69, s
3	74.7, C	-	86.0, C	-
4	37.2, CH_2_	1.83, m;	32.2, CH_2_	2.12, m
5	25.7, CH_2_	1.81, m	36.4, CH_2_	2.68, m
	-	1.90, m	-	2.28, m
6	70.6, CH	5.48, d (8.8) ^d^	206.5, CH	-
7	139.4, C	-	147.7, C	-
8	128.4, CH	5.28, s	37.3, CH_2_	3.22, dd (13.2, 5.6)
	-	-	-	2.34, m
9	78.6, CH	4.47, d (6.0)	78.4, CH	4.08, m
10	54.9, CH	2.70, t (7.2)	48.8, CH	3.08, dd (9.6, 7.6)
11	83.0, C	-	145.2 , C	-
12	30.4, CH_2_	1.32, m	31.2, CH_2_	2.08, m
	-	1.52, m	-	2.27, m
13	18.4, CH_2_	1.35, m	25.9, CH_2_	1.10, m
	-	1.45, m	-	1.65, m
14	42.1, CH	1.26, m	37.5, CH	1.66, m
15	27.7, CH_3_	1.18, s	22.7, CH_3_	1.48, s
16	17.9, CH_3_	1.79, s	118.4, CH_2_	5.27, s
	-	-	-	5.62, s
17	23.7, CH_3_	1.53, s	111.6, CH_2_	4.72, s
	-	-	-	4.85, s
18	29.2, CH	1.72, m	36.4, CH	1.78, m
19	16.5, CH_3_	0.83, d (7.2)	16.3, CH_3_	0.79, d (7.2)
20	21.9, CH_3_	0.96, d (7.2)	66.4, CH_2_	3.52, d (7.2)
11-OAc	22.6, CH_3_	1.99, s	-	-
	170.2, C	-	-	-
2-butanoyloxybutanoate	-	-	-	-
1′	-	-	169.0, C	-
2′	-	-	73.6, CH	4.76, t (6.8)
3′	-	-	24.5, CH_2_	1.83, m
4′	-	-	9.7, CH_3_	1.03, t (7.2)
1′′	-	-	173.5, C	-
2′′	-	-	35.8, CH_2_	2.40, m
3′′	-	-	18.3, CH_2_	1.66, m
4′′	-	-	13.6, CH_3_	0.98, t (7.2)

^a^ Spectra recorded at 100 MHz in CDCl_3_; ^b^ Multiplicity deduced from DEPT; ^c^ Spectra recorded at 400 MHz in CDCl_3_.

Cytotoxicity of compounds **1**–**7** against the proliferation of a limited panel of cancer cell lines, including P388 (murine leukemia), K562 (human erythro myeloblastoid leukemia), A549 (human lung adenocarcinoma), and HT-29 (human colon adenocarcinoma), was evaluated. Compound **5** was found to exhibit cytotoxicity toward P388 and K562 cell lines with IC_50_ values of 13.8 and 36.3 μM (Table 3). Compound **7** displayed cytotoxicity toward A549 cell line with IC_50_ value of 37.2 μM. Other metabolites were found to be inactive against the four cancer cells. The neutrophil pro-inflammatory responses to compounds **1**–**7** were evaluated by suppressing *N*-formyl-methionyl-leucyl-phenylalanine/cytochalasin B (fMLP/CB)-induced superoxide anion (O_2_^•−^) generation and elastase release in human neutrophils, as shown in Table 4. At a concentration of 10 μg/mL, none of compounds were able to significantly reduce the expression of superoxide anion generation, relative to the control cells stimulated with fMLP/CB only. At the same concentration, compound **1** was found to significantly inhibit the elastase release (31.7% ± 3.2% inhibition) in the same fMLP/CB-stimulated neutrophils.

**Table 3:** Cytotoxicity (IC_50_ μM) of compounds **5** and **7**.

Compound	P388	K562	HT-29	A-549
**5**	13.8	36.3	(–) ^a^	(–)
**7**	(–)	(–)	(–)	37.2
5-Fluorouracil	8.5	24.6	20.8	38.5

^a^ IC_50_ > 40 μM.

**Table 4:** Effect of compounds **1**–**7** on superoxide anion generation and elastase release in fMLP/CB-induced human neutrophils at 10 μg/mL.

Compounds	Superoxide Anion		Elastase Release		
	IC_50_ (μg/mL) ^a^	Inhibition %	IC_50_ (μg/mL) ^a^	Inhibition %	
1	>10	1.0 ± 5.5	>10	31.7 ± 3.2	***
2	>10	9.6 ± 5.5	>10	11.5 ± 5.0	-
3	>10	1.7 ± 0.7	>10	17.9 ± 6.9	*
4	>10	6.1 ± 2.6	>10	6.4 ± 2.4	-
5	>10	6.5 ± 2.9	>10	13.6 ± 4.9	*
6	>10	1.0 ± 1.9	>10	6.1 ± 5.6	-
7	>10	4.2 ± 3.8	>10	3.1 ± 6.9	-

Percentage of inhibition (Inh %) at 10 μM concentration. Results are presented as mean ± S.E.M. (*n* = 3 or 4). * *p* < 0.05, ** *p* < 0.01, *** *p* < 0.001 compared with the control value. ^a^ Concentration necessary for 50% inhibition (IC_50_).

## 3. Experimental Section

### 3.1. General Experimental Procedures

Silica gel (230–400 mesh, Merck, Darmstadt, Germany) was used for column chromatography. Precoated silica gel plates (Merck, Kieselgel 60 F-254, 0.2 mm) were used for analytical TLC. High-performance liquid chromatography was performed on a Hitachi L-7100 HPLC apparatus with a Hitachi L-2455 HPLC apparatus (Hitachi Ltd., Tokyo, Japan) with a Supelco C18 column (250 × 21.2 mm, 5 μm). NMR spectra were recorded on a Varian 400MR FT-NMR instrument (Varian Inc, Palo Alto, CA, USA) at 400 MHz for ^1^H and 100 MHz for ^13^C in CDCl_3_. LRMS and HRMS were obtained by ESI on a Bruker APEX II mass spectrometer (Bruker, Bremen, Germany). Optical rotations were measured on a JASCO P-1020 polarimeter. IR spectra were recorded on a JASCO FT/IR-4100 infrared spectrophotometer (Japan Spectroscopic Corporation, Tokyo, Japan).

### 3.2. Animal Material

The animal *Cladiella* *hirsuta* was collected by hand using SCUBA off the coast of Sianglu Islet (23°32' N, 119°38' E) in the region of Penghu Islands, in June 2008, at a depth of 10 m, and was stored in a freezer until extraction. A voucher sample (PI-20080610-17) was deposited at the Department of Marine Biotechnology and Resources, National Sun Yat-sen University.

### 3.3. Extraction and Separation

The frozen bodies of *C.* *hirsuta* (3.1 kg, wet wt) were sliced and exhaustively extracted with acetone (3 × 10 L). The organic extract was concentrated to an aqueous suspension and was partitioned between ethyl acetate (EtOAc) and H_2_O. The EtOAc layer was dried with anhydrous Na_2_SO_4_. After removal of solvent in vacuo, the residue (32.8 g) was subjected to column chromatography on silica gel and eluted with EtOAc in *n*-hexane (0%–100% of EtOAc, gradient) and further with MeOH in EtOAc of increasing polarity to yield 25 fractions. Fraction 18, eluting with *n*-hexane–EtOAc (1:1), was rechromatographed over a Sephadex LH-20 column using acetone as the mobile phase to afford four subfractions (A1–A4). Subfractions A3 and A4 were combined and separated by reversed-phase HPLC (MeOH–H_2_O, 3:1 and 2:1) to afford compounds **4** (1.8 mg), **5** (1.4 mg), **6** (27.7 mg) and **7** (5.6 mg), respectively. Fraction 19, eluting with *n*-hexane–EtOAc (1:2), was rechromatographed over a Sephadex LH-20 column, using acetone as the mobile phase, to afford four subfractions (B1–B4). Subfractions B2 and B3 were combined and separated by reversed-phase HPLC (acetonitrile–H_2_O, 3:1 and 2:1) to afford compounds **1** (9.2 mg), **2** (4.0 mg), and **3** (1.8 mg), respectively.

Hirsutalin N (**1**): colorless oil; [α]^25^_D_ −98 (*c* 0.54, CHCl_3_); IR (neat) *v*_max_ 3451 and 1733 cm^−1^; ^13^C and ^1^H NMR data (400 MHz; CDCl_3_), see Table 1; ESIMS *m/z* 461 [M + Na]^+^; HRESIMS *m/z* 461.2518 [M + Na]^+^(calcd for C_24_H_38_O_7_Na, 461.2515) (Supplementary Information, Figures S1–S3).

Hirsutalin O (**2**): colorless oil; [α]^25^_D_ −128 (*c* 0.68, CHCl_3_); IR (neat) *v*_max_ 3482 and 1729 cm^−1^; ^13^C and ^1^H NMR data (400 MHz; CDCl_3_), see Table 1; ESIMS *m/z* 445 [M + Na]^+^; HRESIMS *m/z* 445.2564 [M + Na]^+^(calcd for C24H38O6Na, 445.2566) (Supplementary Information, Figures S4–S6).

Hirsutalin P (**3**): colorless oil; [α]^25^_D_ +27 (*c* 0.54, CHCl_3_); IR (neat) *v*_max_ 3426 and 1730 cm^−1^; ^13^C and ^1^H NMR data (400 MHz; CDCl_3_), see Table 1; ESIMS *m/z* 435 [M + Na]^+^; HRESIMS *m/z* 435.2720 [M + Na]^+^(calcd for C_23_H_40_O_6_Na, 435.2722) (Supplementary Information, Figures S7–S9).

Hirsutalin Q (**4**): colorless oil; [α]^25^_D_ +12 (*c* 0.51, CHCl_3_); IR (neat) *v*_max_ 3421 and 1724 cm^−1^; ^13^C and ^1^H NMR data (400 MHz; CDCl_3_), see Table 2; ESIMS *m/z* 403 [M + Na]^+^; HRESIMS *m/z*403.2457 [M + Na]^+^(calcd for C22H36O5Na, 403.2460) (Supplementary Information, Figures S10–S12).

Hirsutalin R (**5**): yellow oil; [α]^25^_D_ −18 (*c* 0.54, CHCl_3_); IR (neat) *v*_max_ 3437 and 1740 cm^−1^; ^13^C and ^1^H NMR data (400 MHz; CDCl_3_), see Table 2; ESIMS *m/z* 513 [M + Na]^+^; HRESIMS *m/z* 513.2831 [M + Na]^+^(calcd for C_28_H_42_O_7_Na, 513.2828) (Supplementary Information, Figures S13–S15).

### 3.4. Cytotoxicity Testing

Cell lines were purchased from the American Type Culture Collection (ATCC). Cytotoxicity assays of compounds **1**–**7** were performed using the Alamar Blue assay [32,33].

### 3.5. In Vitro Anti-Inflammatory Assay

Human neutrophils were obtained using dextran sedimentation and Ficoll centrifugation. Measurements of superoxide anion generation and elastase release were carried out according to previously described procedures. [34,35]. LY294002, a phosphatidylinositol-3-kinase inhibitor, was used as a positive control for inhibition of superoxide anion generation and elastase release with IC_50_ 0.6 ± 0.1 and 1.2 ± 0.3 μg/mL [36].

## 4. Conclusions

Five new eunicellin-type compounds, hirsutalins N–R (**1**–**5**) and two known eunicellin-type compounds (**6** and **7**), were discovered from the soft coral *C.* *hirsuta*. Compound **5** displayed cytotoxicity against the proliferation of P388 and K562 cancer cells possibly due to the presence of the α,β-unsaturated ketone group. Compound **1** was found to effectively inhibit the elastase release in FMLP/CB-induced human neutrophils.

## Supplementary Files

Supplementary File 1 Supplementary Information (PDF, 1105 KB)
