# Supplementary material for: Eunicellin-Based Diterpenoids, Hirsutalins N–R, from the Formosan Soft Coral Cladiella hirsuta
Source: Mar Drugs. 2014 Apr 30;12(5):2446–57. doi: 10.3390/md12052446 (PMC4052299; doi:10.3390/md12052446)
Supplement: Supplementary File 1 — Supplementary Information (PDF, 1105 KB) [file marinedrugs-12-02446-s001.pdf]

Supplementary Information

Figure S1. HREIMS spectrum of **1**
Figure S2. ^{1}H NMR spectrum of **1** in $CDCl_3$
Figure S3. ^{13}C NMR spectrum of **1** in $CDCl_3$
Figure S4. HREIMS spectrum of **2**
Figure S5. ^{1}H NMR spectrum of **2** in $CDCl_3$
Figure S6. ^{13}C NMR spectrum of **2** in $CDCl_3$
Figure S7. HREIMS spectrum of **3**
Figure S8. ^{1}H NMR spectrum of **3** in $CDCl_3$
Figure S9. ^{13}C NMR spectrum of **3** in $CDCl_3$
Figure S10. HREIMS spectrum of **4**
Figure S11. ^{1}H NMR spectrum of **4** in $CDCl_3$
Figure S12. ^{13}C NMR spectrum of **4** in $CDCl_3$
Figure S13. HREIMS spectrum of **5**
Figure S14. ^{1}H NMR spectrum of **5** in $CDCl_3$
Figure S15. ^{13}C NMR spectrum of **5** in $CDCl_3$

Figure S1. HREIMS spectrum of **1**.

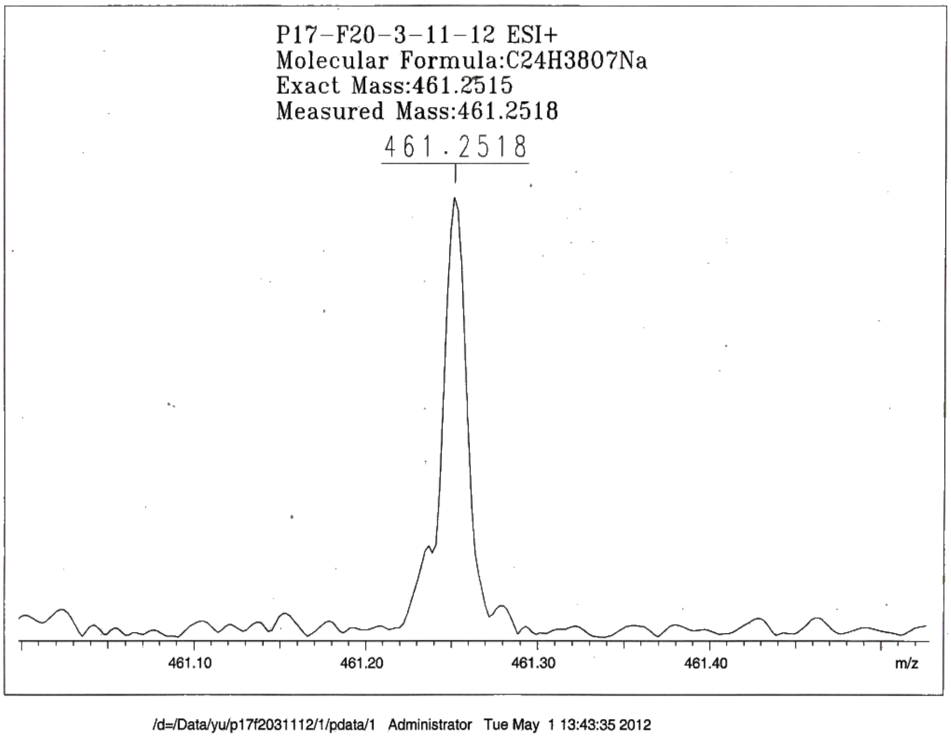

Figure S2. ^{1}H NMR spectrum of **1** in $CDCl_3$.

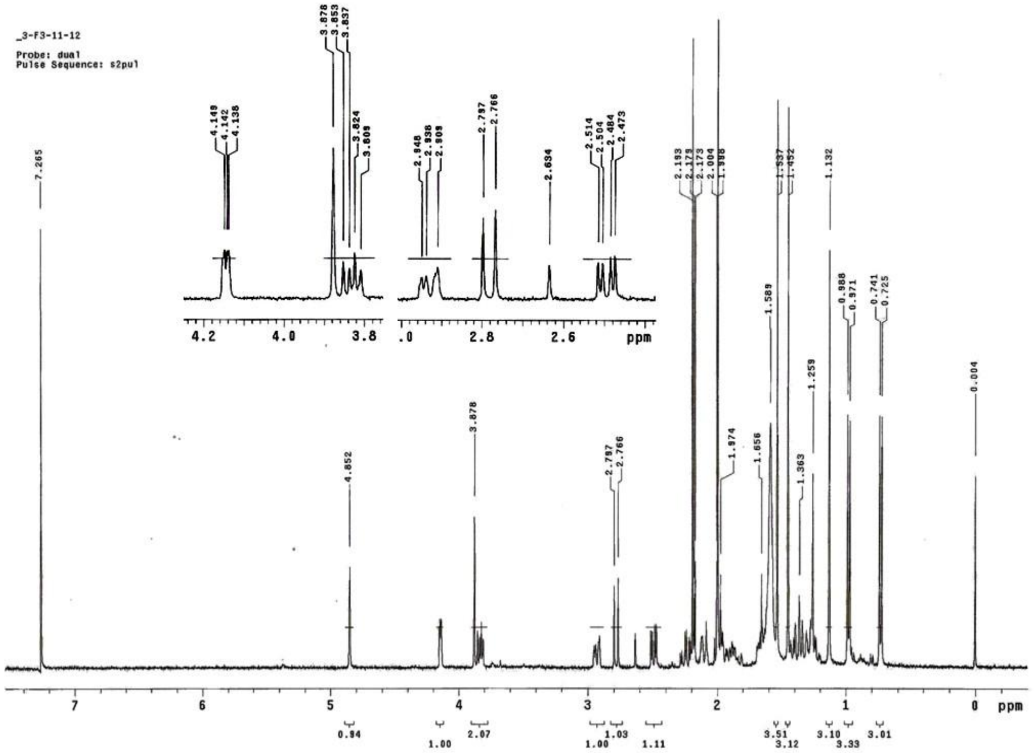

Figure S3. ^{13}C NMR spectrum of 1 in $CDCl_3$.

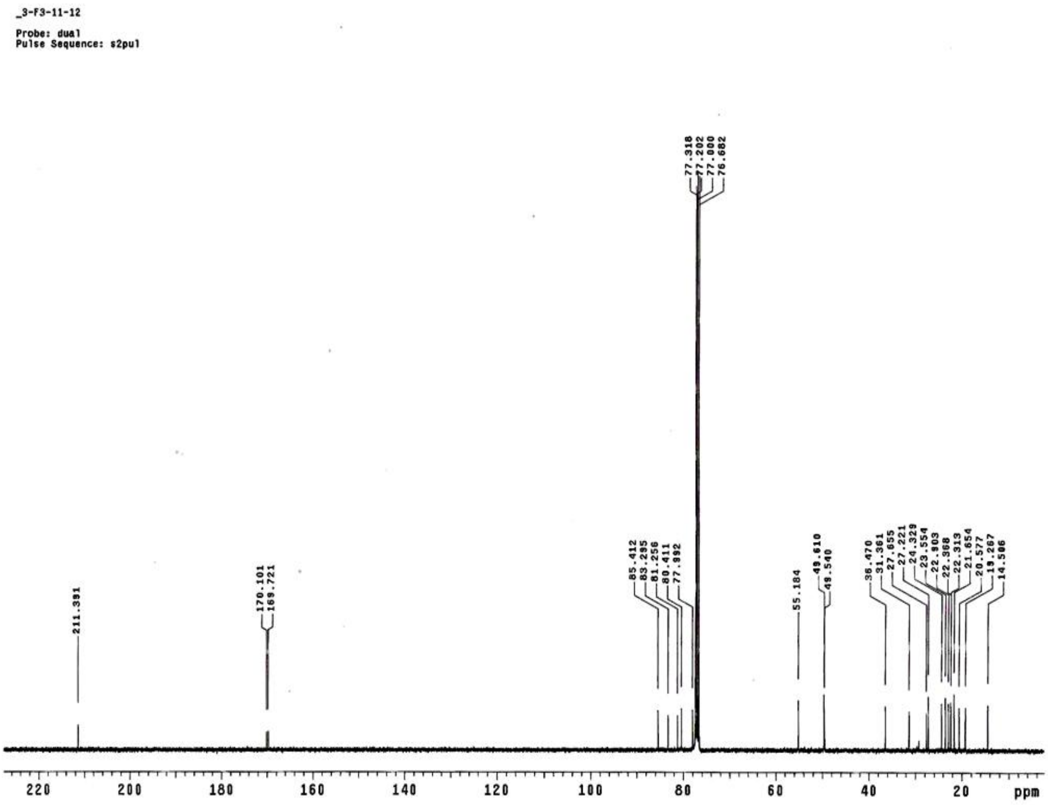

Figure S4. HREIMS spectrum of **2**.

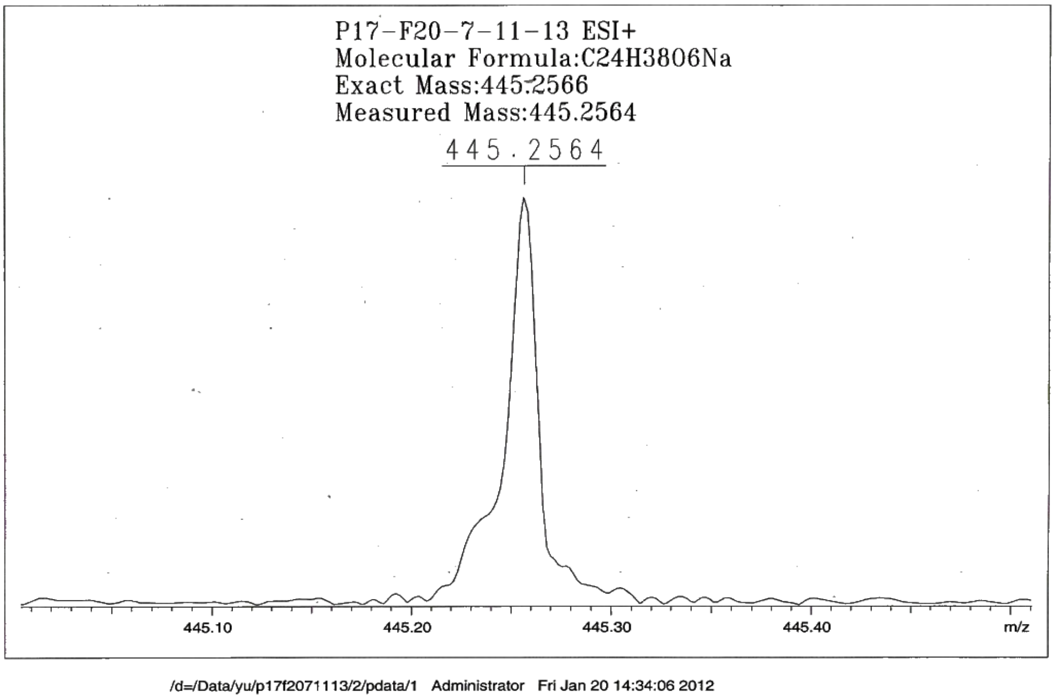

Figure S5. ^{1}H NMR spectrum of **2** in $CDCl_3$.

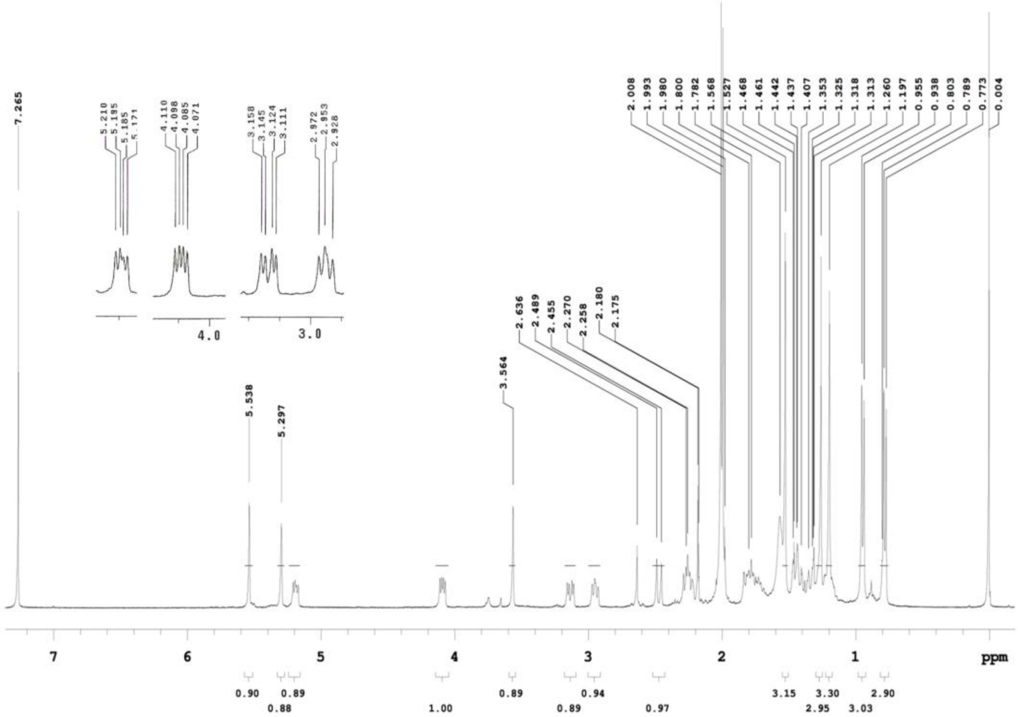

Figure S6. ^{13}C NMR spectrum of 2 in $CDCl_3$.

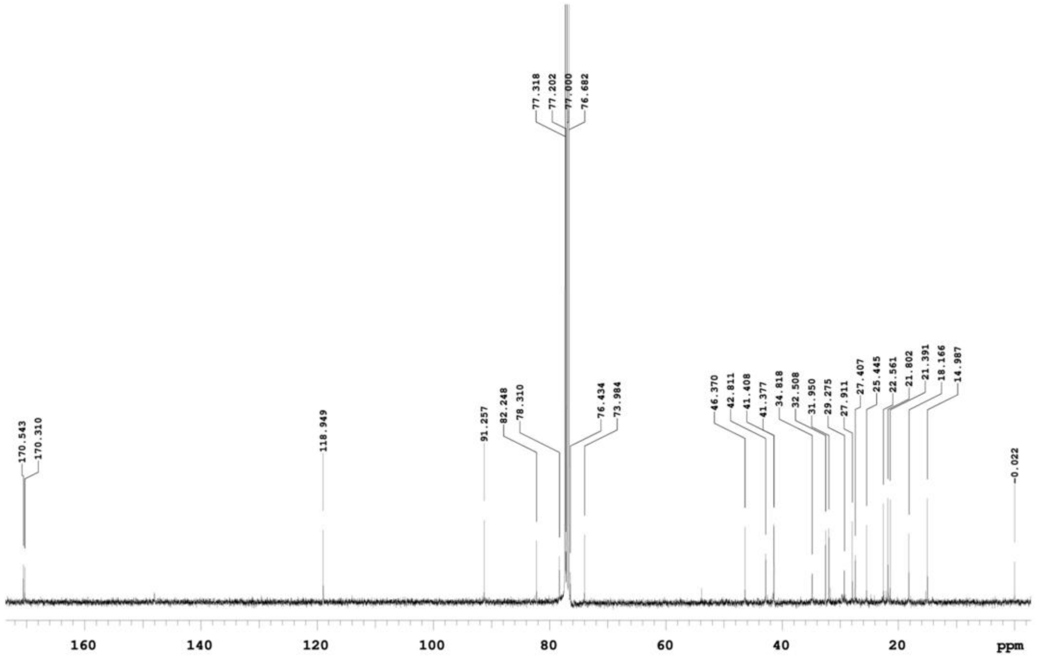

Figure S7. HREIMS spectrum of 3.

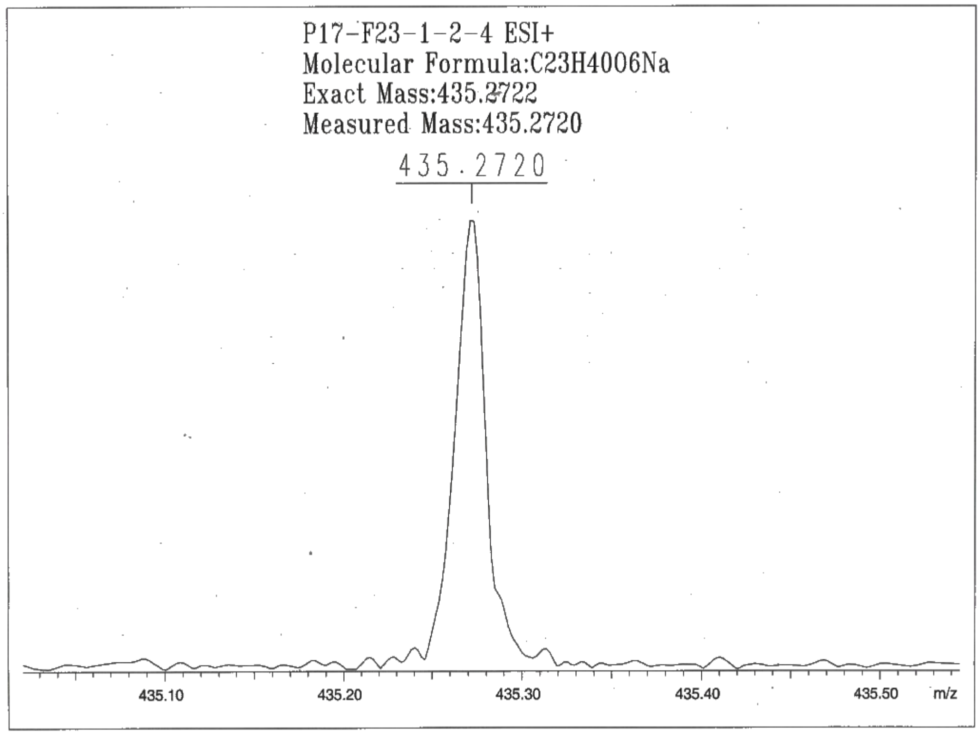

Figure S8. ^{1}H NMR spectrum of **3** in $CDCl_3$.

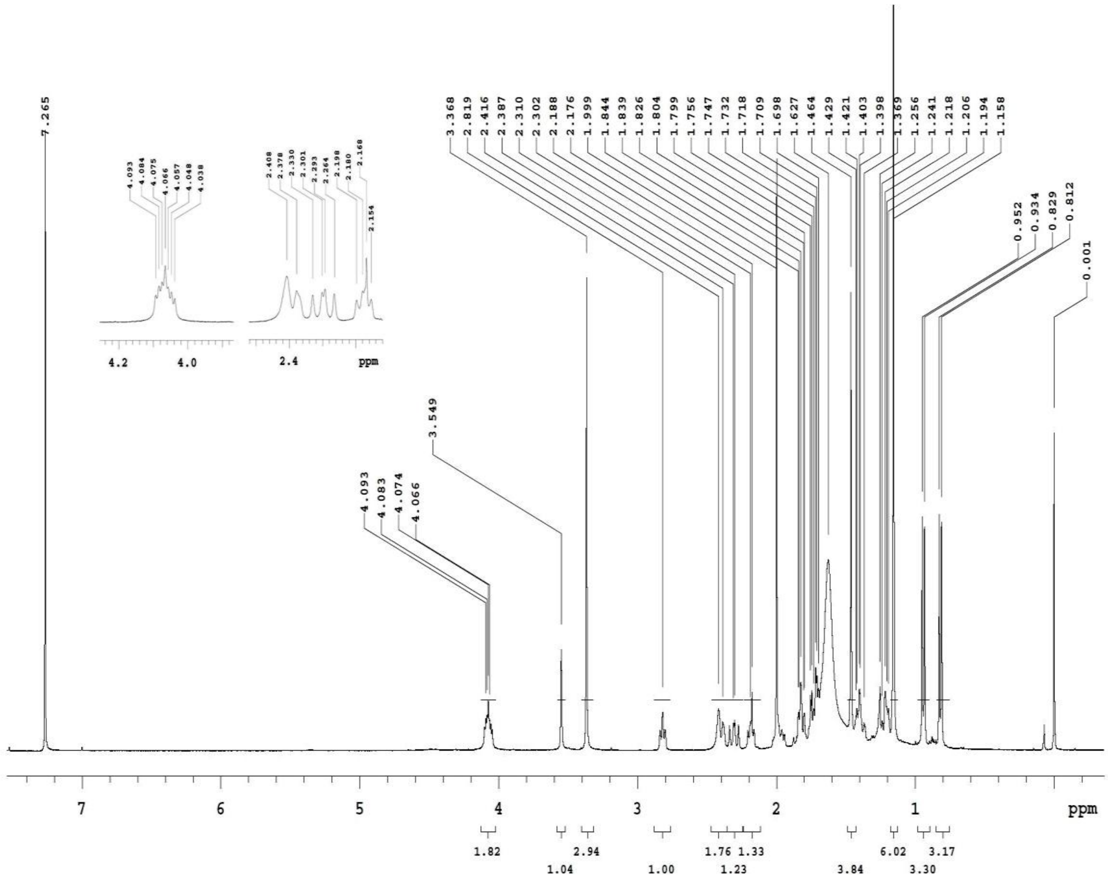

Figure S9. ^{13}C NMR spectrum of **3** in $CDCl_3$.

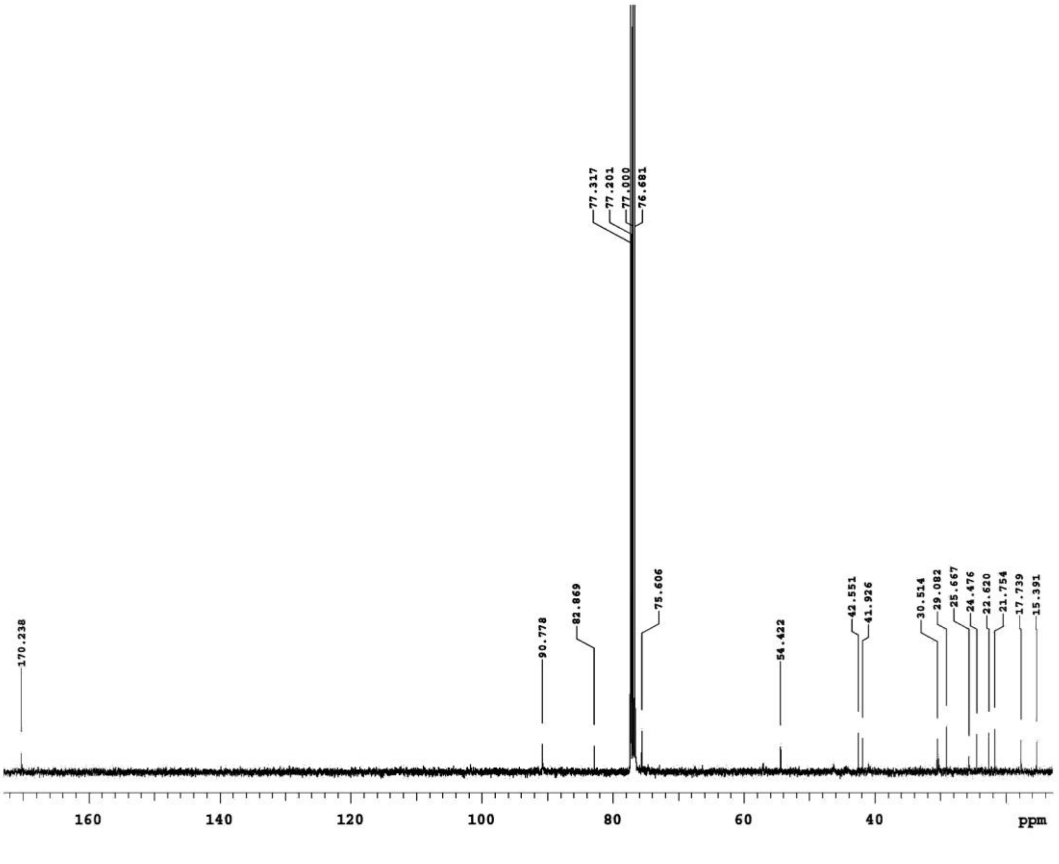

Figure S10. HREIMS spectrum of **4**.

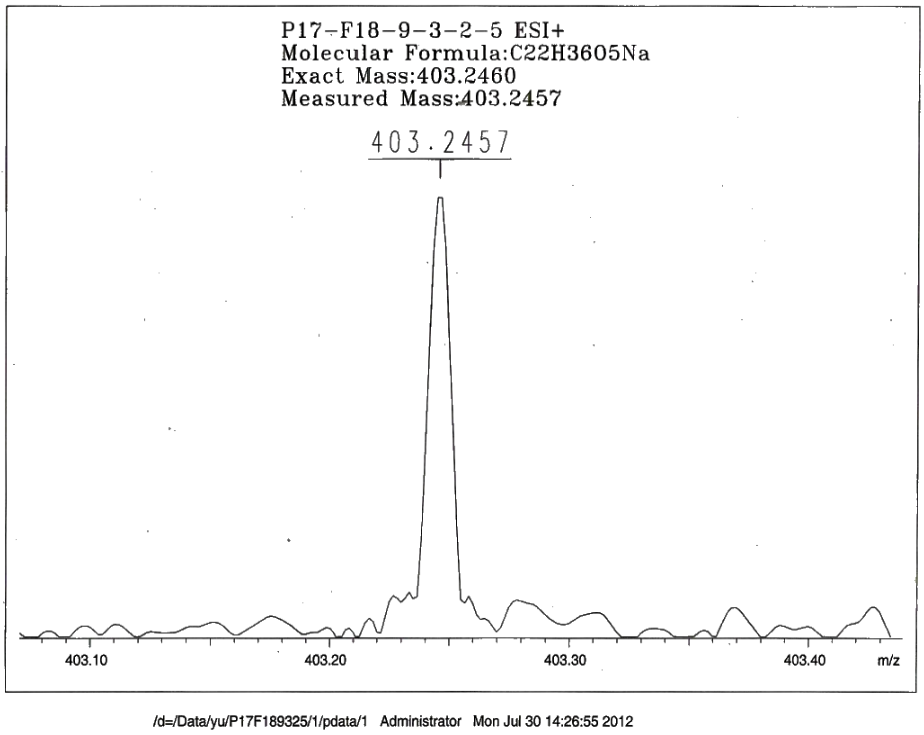

Figure S11. ^{1}H NMR spectrum of 4 in $CDCl_3$.

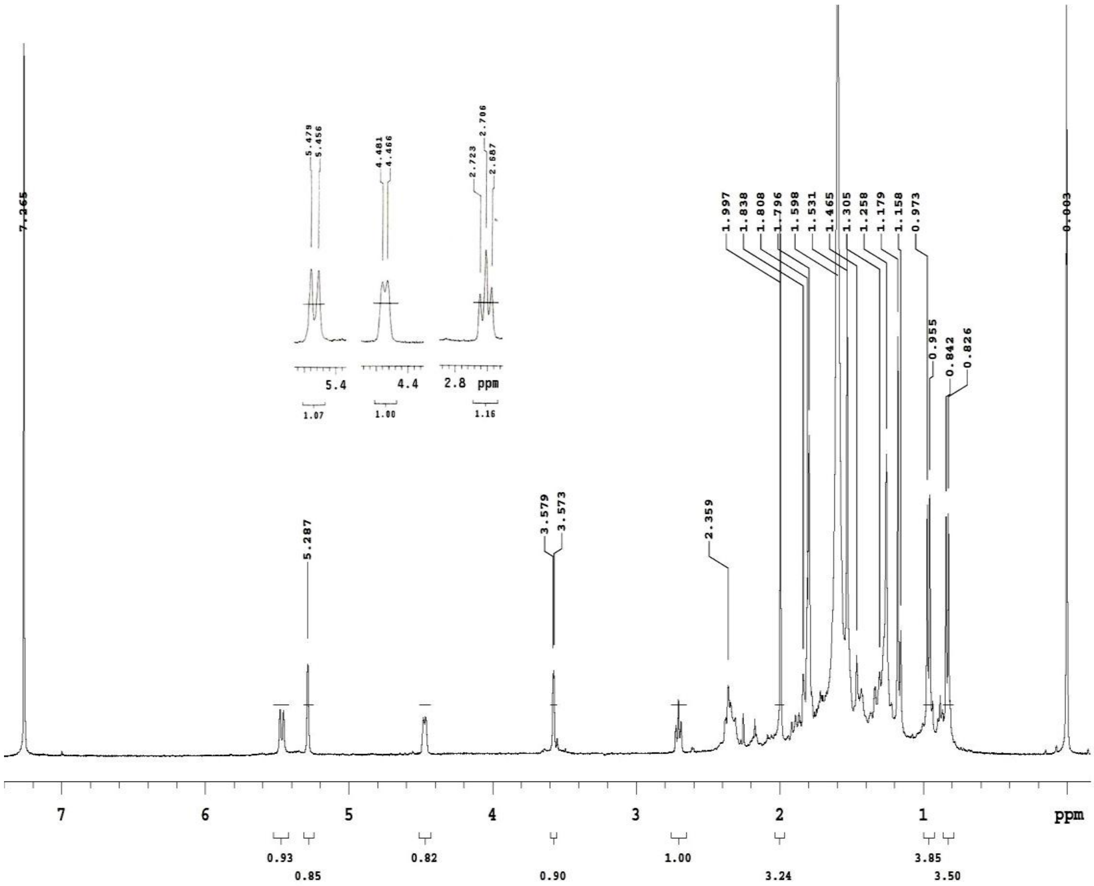

Figure S12. ^{13}C NMR spectrum of **4** in $CDCl_3$.

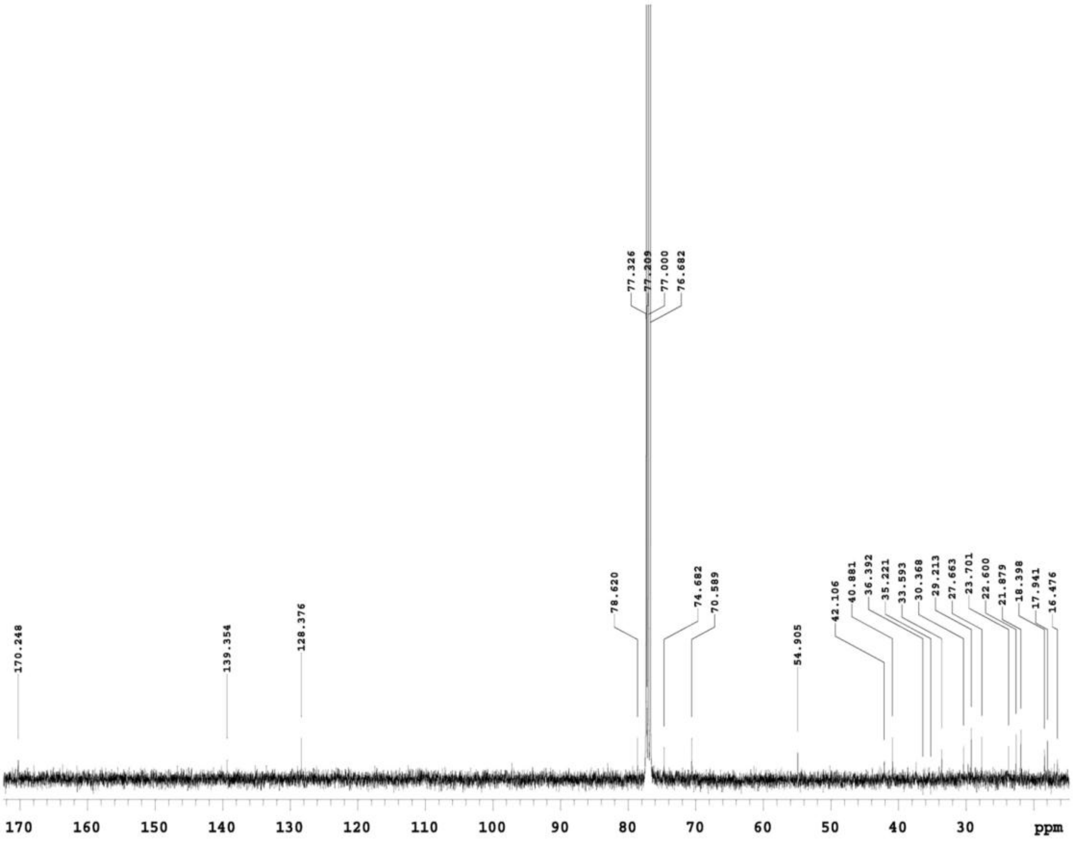

Figure S13. HREIMS spectrum of **5**.

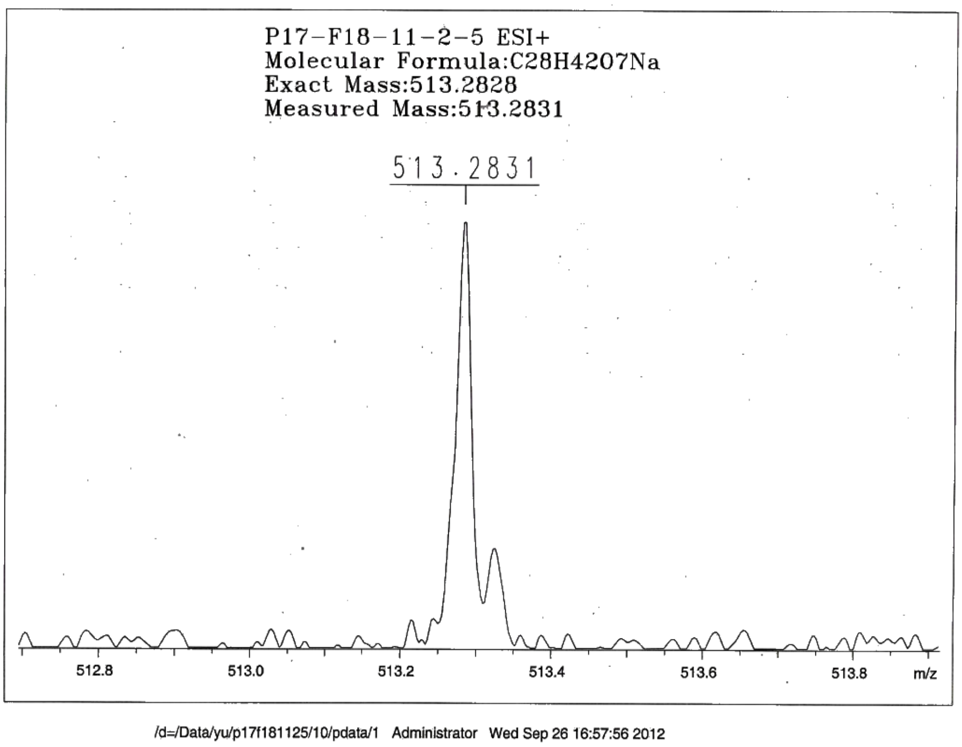

Figure S14. ^{1}H NMR spectrum of 5 in $CDCl_3$.

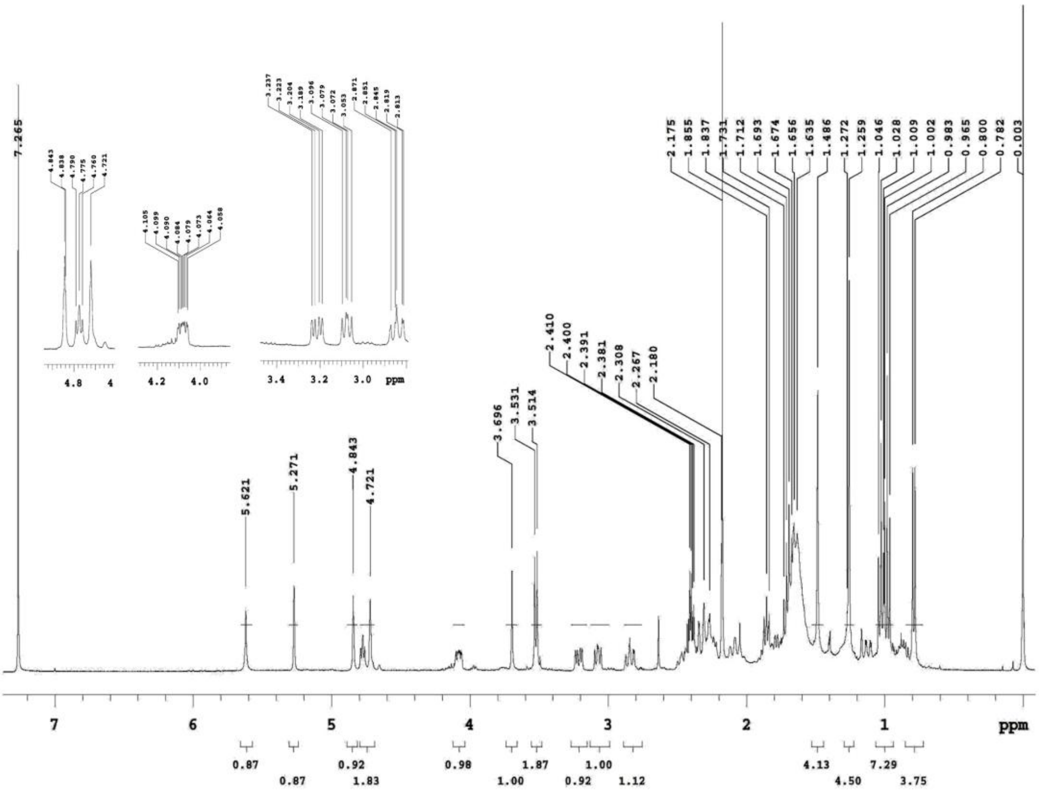

Figure S15. ^{13}C NMR spectrum of **5** in $CDCl_3$**.**

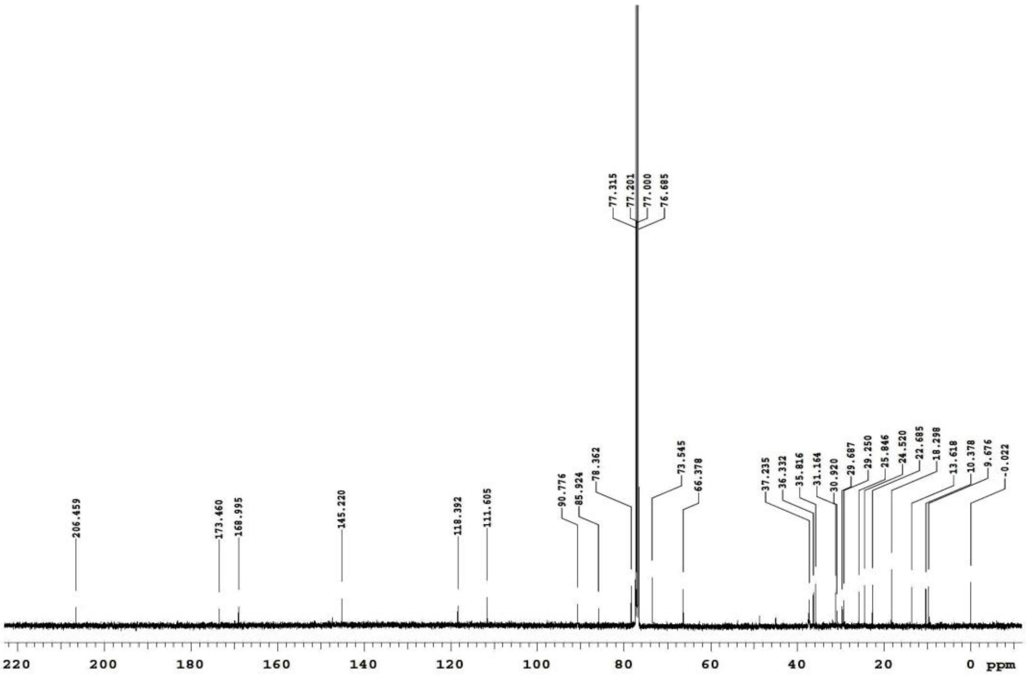

© 2014 by the authors; licensee MDPI, Basel, Switzerland. This article is an open-access article distributed under the terms and conditions of the Creative Commons Attribution license (http://creativecommons.org/licenses/by/3.0/).
